# Prevalence of unmet supportive care needs reported by individuals ever diagnosed with cancer in Australia: a systematic review to support service prioritisation

**DOI:** 10.1007/s00520-023-08146-y

**Published:** 2023-11-07

**Authors:** Jackie Roseleur, Laura Catherine Edney, Jayda Jung, Jonathan Karnon

**Affiliations:** Flinders Health and Medical Research Institute, College of Medicine and Public Health, Flinders University, Adelaide, SA Australia

**Keywords:** Unmet needs, Supportive care needs, Australia, Oncology, Health service needs and demand

## Abstract

**Purpose:**

Improved health outcomes for individuals ever diagnosed with cancer require comprehensive, coordinated care that addresses their supportive care needs. Implementing interventions to address these is confounded by a lack of evidence on population needs and a large pool of potential interventions. This systematic review estimates the point prevalence of different supportive care needs stratified by the tool used to measure needs and cancer type in Australia.

**Methods:**

We searched MEDLINE, Embase, and Scopus from 2010 to April 2023 to identify relevant studies published on the prevalence of supportive care needs in Australia.

**Results:**

We identified 35 studies that met the inclusion criteria. The highest prevalent unmet need across all cancers was ‘fear of cancer spreading’ (20.7%) from the Supportive Care Needs Survey Short-Form 34 (SCNS-SF34), ranging from 9.4% for individuals ever diagnosed with haematological cancer to 36.3% for individuals ever diagnosed with gynaecological cancer, and ‘concerns about cancer coming back’ (17.9%) from the Cancer Survivors’ Unmet Needs (CaSUN), ranging from 9.7% for individuals ever diagnosed with prostate cancer to 37.8% for individuals ever diagnosed with breast cancer. Two studies assessed needs in Aboriginal and Torres Strait Islander populations, reporting the highest needs for financial worries (21.1%).

**Conclusions:**

Point prevalence estimates presented here, combined with estimates of the costs and effects of potential interventions, can be used within economic evaluations to inform evidence-based local service provision to address the supportive care needs of individuals ever diagnosed with cancer.

**Implications for Cancer Survivors:**

Local health services can use local evidence to prioritise the implementation of interventions targeted at unmet needs.

**Supplementary Information:**

The online version contains supplementary material available at 10.1007/s00520-023-08146-y.

## Introduction

Cancer is a leading cause of disease burden, accounting for 250 million disability-adjusted life-years lost internationally in 2019 [1]. An estimated 151,000 Australians will be diagnosed with cancer in 2021, with over 1 million diagnosed in their lifetime [2]. This is expected to rise due to increasing prevalence from population growth, ageing [3], and increased survival due to earlier detection [4] and medical advancements [5]. As a result, more people are living with the effects of cancer. These effects can include a range of supportive care needs, including physical, emotional, social, psychological, informational, spiritual, and practical [6] that may result from either anticancer treatment or a cancer diagnosis itself.

A range of tools have been developed to identify these unmet supportive care needs, including generic tools applicable across all patients with any cancer diagnosis [7], tools for patients with specific cancer types (e.g. breast cancer [8, 9], melanoma [10]), for specific population groups [11], or for specific points on the care continuum [12]. Studies using these tools suggest that supportive care needs are not well met in practice, with systematic reviews reporting high unmet needs across daily living, psychological needs, informational needs, psychosocial needs, physical needs, spiritual needs, communication needs, and sexuality needs domains [13, 14]. These unmet supportive care needs contribute to poor quality of life [15–18] and reduced survival [19, 20] and should, therefore, be addressed as part of routine care to improve health and meet the Australian Optimal Care Pathways [21]. Understanding the prevalence of different unmet supportive care needs can identify current gaps in clinical care and help target service prioritisation to address these.

We undertook a systematic review to analyse and review the available evidence on the prevalence of unmet supportive care needs in Australia from a policymaker perspective, to reflect gaps in the reporting of the results of primary studies and to demonstrate how the gaps can be addressed to support priority setting for services to focus on supportive care needs. We add to an earlier systematic review, published in 2019, evaluating the prevalence of supportive care needs in Australia [14] by including studies published since the earlier review (mid-2018), extending the search strategy to include a wider range of study participants by excluding the need for explicit use of the term *survivors* due to the changing definition of this term over time [22] and employing a different methodology to estimate prevalence, disaggregating results by cancer type and supportive care needs tools.

## Methods

### Search strategy

The present search strategy was adapted from Edney et al. [23] to identify the supportive care needs of individuals ever diagnosed with cancer in Australia. The search strategy aimed to identify relevant studies published since 2010 in MEDLINE, Embase, and Scopus and included Medical Subject Headings, Emtree headings, and related text (title and abstract) and keyword searches using terms to describe the population and supportive care need terms. The search was replicated in Google Scholar using keywords with the first 100 results screened. The search strategy aimed to identify the full breadth of supportive care needs by including synonyms for specific needs identified from all domains of needs identified in the *Supportive Care Framework for Cancer Care* [6] and across common tools used to measure needs as reported previously [23]. The search strategy for MEDLINE is presented in Supplementary Table 1. The initial search was conducted in November 2020, updated in July 2021 and again in April 2023.

### Inclusion criteria

This systematic review included quantitative studies conducted in Australia that employed one of four generic validated tools—Supportive Care Needs Survey Short-Form 34 (SCNS-SF34), Cancer Survivors’ Unmet Needs (CaSUN), Supportive Care Needs Assessment Tool for Indigenous People (SCNAT-IP), and Survivors’ Unmet Needs Survey (SUNS) (see Table 1)—to assess the prevalence of unmet needs in adults (aged 18 or over) who had been diagnosed with any type of cancer at any time. Articles that reported on cancer site-specific validated tools, used a study-specific tool, or reported mixed results that could not be separated by the generic tools were excluded. Peer-reviewed primary studies published between 2010 and 2023 were included; conference abstracts, opinion pieces, letters to the editor and qualitative studies on the experiences of patients and their needs were excluded.

**Table 1:** Domains of supportive care needs captured across tools mapped to the domains included in the Supportive Care Framework for Cancer Care [6]

	Unmet supportive care needs tools			
	SCNS-SF34	SCNAT-IP	CaSUN	SUNS
Number of domains	5	4	6	5
Eligible population	Cancer diagnosis	Indigenous Australians with a cancer diagnosis	Cancer diagnosis	1- to 5-years post cancer diagnosis
Number of items	34	39	35	89
Recall period	Prior month	Prior month	Prior month	Prior month
Response levels/rating scale	5	5	5	5
Supportive care needs framework domains [6]				
Psychological	Psychological	Physical and psychological	Quality of life/existential survivorship	
Physical	Physical and daily living	Physical and psychological		
Emotional				Emotional health
Practical		Practical and cultural		Financial concerns
Informational	Health system and information	Information and communication	Information	Information
Spiritual				
Social			Relationships	Relationships
Domains not included in Fitch [6]				
	Patient care and support	Hospital care	Comprehensive cancer care	Access and continuity of care
	Sexuality		Other	

### Data extraction

An extraction form was developed and tested to ensure all relevant information was included in the form. Data were extracted by one reviewer (JJ) and reviewed by a second (JR). Extracted information included bibliographic details, geographical location (state/territory, remoteness), cancer site and stage, time since diagnosis, sample size, patient age and gender, eligibility criteria, study design, tool employed, sample response rate, overall proportion reporting moderate to high unmet supportive care needs, top supportive care needs reported, any sub-group analyses undertaken, and the prevalence of unmet needs. Baseline data was extracted for longitudinal studies and corresponding authors were contacted where complete data were not reported.

### Assessment of methodological quality

The quality of all eligible studies was assessed using a tool for systematic reviews adapted from Mols et al. [24] by Miroševič et al. [25] (see Supplementary Table 2). Standard quality appraisal tools for prevalence studies were not appropriate for measuring supportive care needs here as their focus was on measuring the prevalence of conditions. One question was excluded, which was not relevant to our study aim (Q11: an attempt is made to find factors associated with higher unmet needs), resulting in the highest possible score of 11. Studies scoring more than 7 were considered high quality; studies scoring between 5 and 7 were considered moderate quality, and studies scoring lower than 5 were considered low quality. Studies were included if they met 5 or more of the 11 quality criteria [25].

### Data synthesis

The prevalence of moderate or high unmet needs by broad domains of need (see Table 1) was synthesised by cancer type and by tool employed. Prevalence by cancer was estimated through weighted averages based on sample size for items across studies using the same tool. Not all studies reported the prevalence of all items in the tools, assuming no prevalence for these items represents an underestimate of the true prevalence. We therefore assumed the prevalence of unreported items at 50% of the items with the lowest reported prevalence for each study with incomplete data. Sensitivity analyses were conducted where the prevalence for each unreported item was assumed to be 0 (representing an underestimate of the true prevalence) and at 0.1% below the lowest reported prevalence (representing an overestimate of the true prevalence).

## Results

**Fig. 1:**
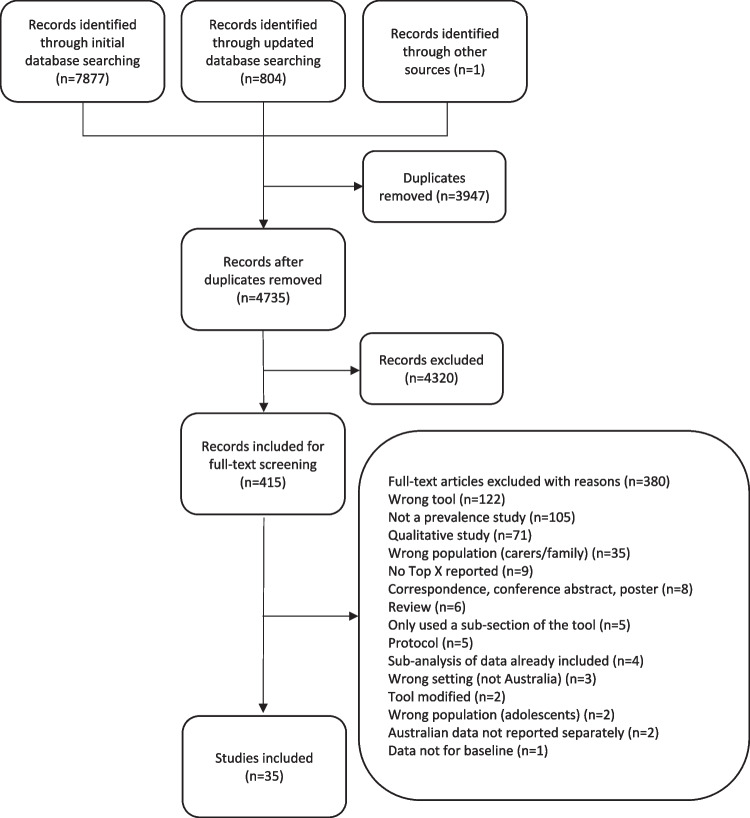
PRISMA diagram of literature search and study selection with reasons for full-text exclusion

A total of 7877 records were identified in the initial search of the three databases, with an additional 211 records identified in the updated search in July 2021 and a further 593 records in April 2023. One author who was contacted for primary data provided an additional study that had not been captured in our search. After removing duplicates, 4735 titles and abstracts were independently screened against the inclusion criteria by two reviewers (JR and LE). The remaining 415 records underwent full-text review (JR and JJ), resulting in 35 records for inclusion. The primary reason for exclusion at full-text review was the use of a tool other than those specified in the inclusion criteria (*n* = 122). Other exclusion reasons are presented in the PRISMA diagram (Fig. 1).

## Characteristics of included studies

Of the 35 studies included, 30 were cross-sectional and five were longitudinal (Table 2), with the majority (71%) published since 2014. Participants ranged from a mean age of 35 [26] through to 76 years [27], with the range of time since diagnosis varying from less than three months [28–31] to more than 20 years [32–34]. A range of cancer types was included: nine studies presented prevalence across different cancer types, one compared breast and brain cancer, and the remaining 25 focused on single types of cancer, including gynaecological (*n* = 7), haematological (*n* = 4), breast (*n* = 5), brain (*n* = 3), prostate (*n* = 3), testicular (*n* = 1), neuroendocrine (*n* = 1), and pancreatic (*n* = 1) cancer. The majority of studies captured unmet needs with SCNS-SF34 (*n* = 21); 10 used CaSUN, two used SUNs, and two used SCNAT-IP. Only 6 of the 35 studies reported prevalence estimates for each item on the tool used. Study authors of the remaining studies were contacted for unreported data, which led to full responses for an additional 14 studies. Incomplete prevalence data for all items remained for 15 studies.

**Table 2:** Characteristics of included studies (*n* = 35)

First author, year	Study design	Tool	Cancer type	Sample size, participant characteristics	Time since diagnosis	Data reported^a^
Ahern (2016) [45]	Cross-sectional	SCNS-SF34	Breast	*n* = 839, mean age = 56, female = 100%	> 6 months post treatment	Partial^d^
Alananzeh (2019) [46]	Cross-sectional	SCNS-SF34	Breast (30.3%), prostate (13.6%), colorectal/bowel (15.3%), lung (10.6%), leukaemia/lymphomas (13.6%)	*n* = 66, mean age = 62, female = 54.5%	47% < 12 months post diagnosis	Partial^d^
Beesley (2013) [47]	Longitudinal	SCNS-SF34	Gynaecological (ovarian only)	*n* = 185, mean age = 59, female = 100%	6–12 months post diagnosis	Partial^e^
Beesley (2016) [29]	Cross-sectional	SCNS-SF34	Pancreatic and ampullary	*n* = 136, mean age = 66, female = 40%	< 10 months post diagnosis (61% 0–3 months post diagnosis)	Partial^e^
Beesley (2018) [32]	Cross-sectional	SCNS-SF34	Neuroendocrine	*n* = 111, 61% aged 60+, female = 44%	2 months to 27 years post diagnosis (35% 2–5 years post diagnosis)	Partial^e^
Blaschke (2019) [48]	Longitudinal	SCNS-SF34	Breast (metastatic only)	*n* = 62, mean age = 60, female = 100%	0–10 years post diagnosis (median = 2 years)	Partial^d^
Boyes (2012) [49]	Cross-sectional	SCNS-SF34	Prostate (26%), melanoma (16%), breast (16%), blood (14%), colorectal (12%), lung (9%), head and neck (7%)	*n* = 1323, median age = 63, female = 41%	4–9 months post diagnosis	Partial^e^
Boyes (2015) [50]	Cross-sectional	SCNS-SF34	Haematological	*n* = 311, 55% aged 55–74, female = 42%	53% > 24 months post diagnosis	Partial^e^
Chambers (2012) [51]	Cross-sectional	SCNS-SF34	Breast (30.7%), colorectal (9.3%), prostate (8.8%), haematological (7.7%), lung (7.7%), gynaecological (7.5%), melanoma (4.1%)	*n* = 354, mean age = 58, female = 82.5%	1.74 years (mean) post diagnosis	Partial^d^
Dunn (2021) [33]	Cross-sectional	SCNS-SF34	Breast (19.3%), skin (12.6%), head and neck (14.3%), prostate (12.3%), other (41.5%)	*N* = 518, mean age = 65, female = 47.3%	1 day to 33.7 years post diagnosis (median = 7 months)	Partial^e^
Eggins (2022) [52]	Cross-sectional	SCNS-SF34	Breast	*n* = 3326, mean age NR, female = 100%	NR	Partial^e^
Gough (2022) [53]	Cross-sectional	SCNS-SF34	Gynaecological	*n* = 311, mean age = 57, female 100%	Before commencing curative radiotherapy	Partial^e^
Halkett (2015) [54]	Cross-sectional	SCNS-SF34	Brain (glioma only)	*n* = 116, mean age = 56, female = 29.3%	Undergoing chemoradiotherapy	Partial^e^
Hall (2012)^b^ [55]	Cross-sectional	SCNS-SF34	Melanoma (36.2%), breast (25.9%), non-Hodgkin’s lymphoma (20.7%), leukemia (10.3%), other (6.9%)	*n* = 58, mean age = 35, female = 70.7%	7 months (mean) post diagnosis	Partial^d^
Hyde (2017) [56]	Cross-sectional	SCNS-SF34	Prostate	*n* = 331, mean age = 65, female = 0%	≤ 12 months post diagnosis	Full
Kusters (2015) [57]	Cross-sectional	SCNS-SF34	Gynaecological	*n* = 160, mean age = 61, female = 100%	5–30 months post diagnosis	Partial^e^
Langbecker (2016) [31]	Longitudinal	SCNS-SF34	Brain	*n* = 40, mean age = NR, female = 42.5%	≤ 3 months post diagnosis	Partial^d^
McDowell (2010) [58]	Longitudinal	SCNS-SF34	Breast (32.6%), haematological (15.7%), gastrointestinal (14.1%), skin (10.3%), head and neck (8.7%), respiratory (6.6%), genitourinary (6.6%), other (5.5%)^c^	*n* = 439, mean age = 59, female = 59%	7 years (mean) post diagnosis	Partial^d^
Oberoi (2017) [59]	Longitudinal	SCNS-SF34	Haematological (diffuse large B cell lymphoma, multiple myeloma)	*n* = 414, mean age = 64, female = 43.5%	7 months (mean) post diagnosis	Partial^d^
Williams (2018) [34]	Cross-sectional	SCNS-SF34	Gynaecological	*n* = 343, 82% aged 35–75, female = 100%	0–22 years post treatment (median = 17 months)	Full
Yates (2021) [30]	Cross-sectional	SCNS-SF34	Prostate	*n* = 51, mean age = 63, female = 0%	92% newly diagnosed	Partial^e^
Bernardes (2021) [35]	Cross-sectional	SCNAT-IP	Digestive (23%), breast (21%), lung (15%), head and neck (13%), blood-related (10%), gynaecological (6%), other (11%)	*n* = 145, 43% aged ≥ 60, female = 57%	69% < 1 year	Full
Garvey (2015) [28]	Cross-sectional	SCNAT-IP	Breast (24.2%); respiratory (13.7%); lymphoid, haematopoietic, and related tissue (12.9%); digestive organs (12.5%); lip, oral cavity, and pharynx (8.9%); male genital organs (7.3%); female genital organs (7.3%); eye, brain, and other parts of the CNS (4.8%); unknown (2%)	*n* = 248, 55% aged 40–59, female = 57%	56% ≤ 3 months post diagnosis	Full
Amatya (2014) [60]	Cross-sectional	CaSUN	Breast (45%), brain (55%)	Breast: *n* = 85, mean age = 58, female = 100% Brain: *n* = 106, mean age = 53, female = 58%	Breast, 2.2 years (median) post diagnosis Brain, 2.1 years (median) post diagnosis	Partial^d^
Brennan (2016)[61]	Cross-sectional	CaSUN	Breast	*n* = 68, mean age = 56; female = 100%	NR	Partial^e^
Khan (2013) [62]	Cross-sectional	CaSUN	Brain	*n* = 106, mean age = 51, female = 56%	2.1 years (median) post diagnosis	Partial^d^
Mazariego (2020) [27]	Cross-sectional	CaSUN	Prostate	*n* = 351, mean age = 76, female = 0%	15 years	Partial^d^
Molassiotis (2017) [63]	Cross-sectional	CaSUN	Multinational study on different cancer types (NR for Australia)	*n* = 103, mean age = 60, female = 50%	NR. Completed first-line treatment	Partial^e^
Rowlands (2015) [64]	Cross-sectional	CaSUN	Gynaecological (endometrial only)	*n* = 629, 55% aged 50–65, female = 100%	3–5 years post diagnosis	Partial^d^
Smith (2013) [65]	Cross-sectional	CaSUN	Testicular	*n* = 244, mean age = 38, female = 0%	0.5–5 years post treatment completion	Partial^d^
Stafford (2011) [66]	Cross-sectional	CaSUN	Gynaecological	*n* = 176, mean age = 59, female = 100%	4.7 years (mean) post diagnosis	Partial^d^
Urbaniec (2011) [67]	Cross-sectional	CaSUN	Gynaecological	*n* = 45, mean age = 57, female = 100%	0.9–11.6 years post diagnosis	Partial^d^
Vuksanovic (2021) [68]	Cross-sectional	CaSUN	Breast	*n* = 130, 55% aged 46–65, female = 100%	37.3 months (mean) post diagnosis	Partial^e^
Hall (2015) [26]	Cross-sectional	SF-SUNS	Haematological	*n* = 715, 55% aged 60–80 at diagnosis, female = 41%	35 months (median) post diagnosis	Full
Tzelepis (2018) [69]	Cross-sectional	SF-SUNS	Haematological	*n* = 1511, mean age = 58, female = 43%	3.4 years (mean) post diagnosis	Full

^a^‘Full’ means that the prevalence for all items on the screening tool was reported by the study authors. ‘Partial’ means that the prevalence for only some of the items from the screening tool was reported by the study authors

^b^Two populations were reported (18–40 years and 64+ years); the older age group was excluded because the complete survey tool for the SCNS was not used

^c^Reported in Tzelepis et al. [69]

^d^Corresponding author contacted; additional prevalence of unreported items not provided

^e^Corresponding author contacted; additional prevalence of unreported items provided

*CaSUN* Cancer Survivors’ Unmet Needs, *NR* not reported, *SCNAT-IP* Supportive Care Needs Assessment Tool for Indigenous People, *SCNS-SF34* Supportive Care Needs Survey Short-Form 34, *SF-SUNS* Short-Form Survivor Unmet Needs Survey, *NR* not reported

## Methodological quality

Most studies (26/35) were high quality (Supplementary Table 3), and all met the minimum requirement for methodological quality [25]. All studies presented inclusion and exclusion criteria and employed a standardised or validated unmet needs measure due to our inclusion criteria. Most described informed patient consent (33/35), socio-demographic profile of the sample (34/35), type of cancer treatment (31/35), time since diagnosis or treatment (24/35), and study limitations (32/35). Less than a fifth (5/35) reported participation and response rates over 75%.

## The most prevalent unmet needs of cancer patients in Australia across all cancers

### Supportive Care Needs Survey Short-Form 34 (SCNS-SF34)

Table 3 presents the top ten unmet supportive care needs reported by cancer survivors from studies using the SCNS-SF34. In this table, the findings are presented by cancer type for the studies that only included participants with specific cancers (*n* = 15 studies) and by mixed cancer type for the studies that included patients with various cancers (*n* = 6 studies). The last column presents the aggregate results across all 21 studies. Prevalence estimates for all 34 items are provided in Supplementary Table 4.

**Table 3:**
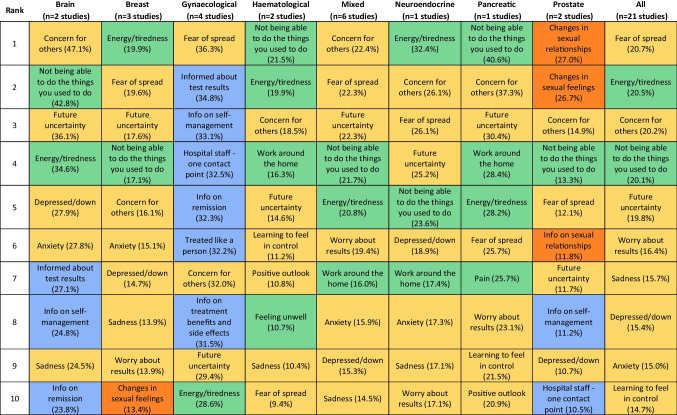
Prevalence of the top 10 unmet supportive care needs as identified by the Supportive Care Needs Survey Short-Form 34 (SCNS-SF34) by cancer type, studies including all cancer types (mixed) and aggregated across all studies

Items are colour-coded by domains. SCNS-SF has five domains (no items from patient care and support needs): green, physical and daily living needs; blue, health system and information needs; orange, sexual needs; and yellow, psychological needs

#### Aggregated results across all cancer types

The aggregated results found that the highest prevalence was observed for items relating to psychological needs, with prevalence estimates ranging from 14.7 to 20.7%. Among the top 10 unmet supportive care needs, the remaining items were related to physical and daily living needs, specifically, changes in usual activities and energy/tiredness.

#### Results by cancer type

When examining studies grouped by cancer type, including mixed cancer type, it becomes evident that more than half of the top 10 prevalent unmet supportive care needs fell within the psychological needs domain (indicated in yellow). This is followed by the domain of physical and daily living needs (represented in green), which accounts for a quarter of the top 10 prevalent unmet needs. Notably, concern for others and future uncertainty ranked among the top 10 for all cancer types, with the highest prevalence reported by individuals ever diagnosed with brain cancer (47.1%) and pancreatic cancer (37.3%) and the lowest prevalence observed in individuals ever diagnosed with prostate cancer (14.9%, 11.7%). Gynaecological and prostate cancers were the only two cancer types where less than half of the top 10 prevalent items were within the psychological needs domain. Those ever diagnosed with gynaecological cancer had high health system and information needs. In contrast, those ever diagnosed with prostate cancer had high sexual needs. Sexual needs were exclusively prevalent in the top 10 among those ever diagnosed with prostate or breast cancer.

### Cancer Survivors’ Unmet Needs (CaSUN)

The top ten unmet supportive care needs, as measured by the CaSUN tool, are presented in Table 4.

**Table 4:**
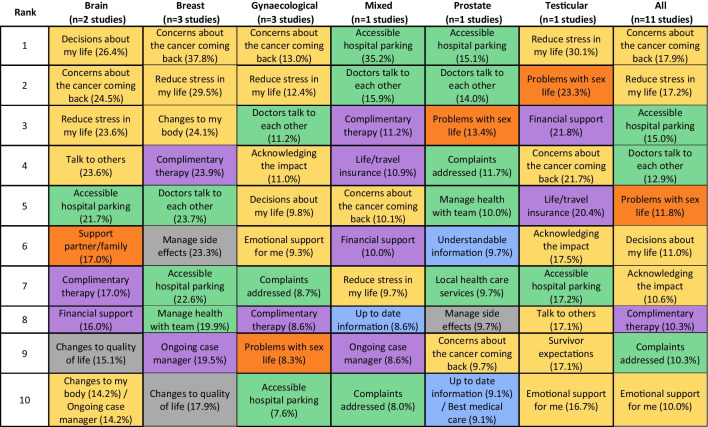
Prevalence of the top 10 unmet supportive care needs as identified by the Cancer Survivors’ Unmet Needs (CaSUN) tool by cancer type, studies including all cancer types (mixed) and aggregated across all studies

Items are colour-coded by domains. CaSUN has six domains: yellow, existential survivorship; grey, quality of life; orange, relationships; green, comprehensive cancer care; blue, information; purple, other

#### Aggregated results across all cancer types

Aggregate results reported that half of the items with the highest prevalence were related to psychological needs, with prevalence estimates ranging from 10.0 to 17.9%. Comprehensive cancer care, sexual needs, and complementary therapy were also identified at the aggregate level. Prevalence estimates for all 35 items are provided in Supplementary Table 5.

#### Results by cancer type

For breast, brain, testicular, and gynaecological cancer, three or more of the top ten items belong to the existential survivorship domain. In contrast, prostate cancer and studies with mixed cancer types reported fewer items in the existential survivorship domain, reporting high needs for items from the comprehensive cancer care domain. Concerns about the cancer coming back was ranked in the top 10 for all cancers, with prevalence ranging from 37.8% for breast cancer patients to 9.7% for prostate cancer patients. Accessible hospital parking was also ranked in the top 10 for all cancers, and the prevalence ranged from 35.2% for studies with different cancer types to 7.6% for gynaecological cancer patients.

### Survivors’ Unmet Needs Survey (SUNS)

Four studies reported on the unmet needs of individuals ever diagnosed with haematological cancer: two captured unmet needs using SUNS and two used SCNS-SF34 to capture unmet needs, as reported in Table 2. A comparison of the top 10 reported unmet needs captured with each tool is reported in Table 5. The most prevalent needs identified were psychological or emotional needs, regardless of the tool used. SCNS-SF34 additionally identified several needs in the physical health domain, including ‘not being able to do the things you used to do’, ‘energy/ tiredness’, ‘work around the home’, and ‘feeling unwell’. These items are similar to items reported in the emotional health domain in the SUNS tool, though these are framed as the psychological impact of dealing with these physical changes.

**﻿Table 5:**
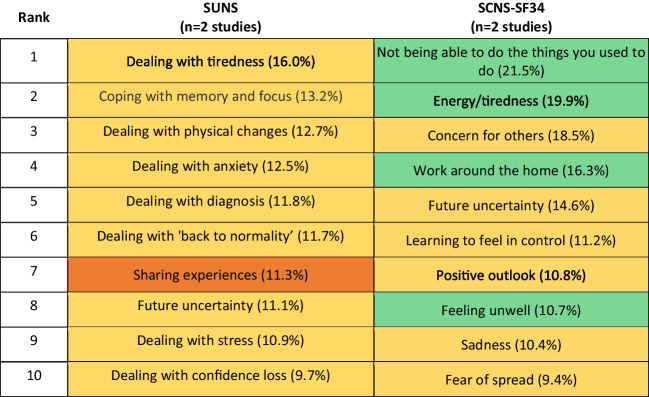
Prevalence of the top 10 unmet supportive care needs for individuals diagnosed with haematological cancer as identified by the Survivors’ Unmet Needs Survey (SUNS) compared to the Supportive Care Needs Survey Short-Form 34 (SCNS-SF34)

Items are colour-coded by domains. SUNS has five domains; SCNS-SF34 has five domains (SUNS, no items from financial concerns, information, access, and continuity of care; SCNS-SF34, no items from health system and information, patient care and support, sexuality): SUNS colour codes: yellow, emotional health; orange, relationships; SCNS-SF34 colour codes: yellow, psychological needs; green, physical and daily living needs

## Prevalence rankings of unmet needs of Aboriginal and Torres Strait Islander cancer patients

### Supportive Care Needs Assessment Tool for Indigenous People (SCNAT-IP)

Two studies assessed unmet needs specifically in Aboriginal and Torres Strait Islander populations using the SCNAT-IP. The first study [28] had 55% of participants aged between 40 and 59, with 55% diagnosed within the last 3 months, whilst the second study [35] had a median age of 57 years (interquartile range of 47.5–64.5) with 70% diagnosed in the last year. Both studies had a similar proportion of female participants, with 57% in each study, and included patients with any cancer. The most prevalent unmet need reported by Aboriginal and Torres Strait Islanders was financial worries, such as the cost of accommodation and travel for treatment. As with the tools designed for the general population, the unmet needs with some of the highest prevalence were in the physical and psychological needs domain, with the common theme of uncertainty and worrying around the recurrence of cancer (see Table 6). Prevalence estimates for all 26 items are provided in Supplementary Table 6.

**Table 6:**
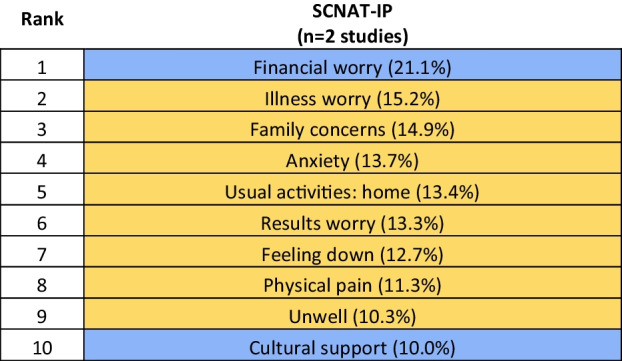
Prevalence of the top 10 unmet supportive care needs as identified by the Supportive Care Needs Assessment Tool for Indigenous People (SCNAT-IP)

Items are colour-coded by domains. SCNAT-IP has four domains (no items from: Information and communication; hospital care): blue, practical and cultural need; yellow, physical and psychological need

## Discussion

This review sought to estimate the prevalence of supportive care needs in Australians ever diagnosed with cancer. Our goal was to identify evidence gaps and, based on the most prevalent unmet needs, inform research priorities and service planning for supportive care needs. An extensive literature review identified a total of 35 primary quantitative studies. The highest unmet supportive care needs fell in the domain of psychological needs, including items related to fear of cancer spreading or recurring, concern for others, and stress, followed by items in the physical need domain. This high prevalence of psychological needs followed by physical needs was similarly reported in a 2019 review [14], despite the inclusion here of an additional 20 primary studies due to broader inclusion criteria, the exclusion of studies prior to 2010, and the use of a different methodology to estimate prevalence. However, the actual prevalence reported differed between the two reviews, with point estimates reported herein toward the lower bound of the ranges reported in Lisy et al. [14]. Whilst Lisy et al. [14] report the lowest and highest prevalence reported across primary studies where the specific item was endorsed by more than 10% of the study, we estimated a weighted average prevalence across all primary studies stratified by cancer type. For example, whilst fear of cancer recurrence was identified as a high need in both reviews, the reported prevalence in Lisy et al. [14] ranged from 14 to 42%, whereas we report a weighted average prevalence of 20.7% based on SCNS-SF34 and 17.9% based on CaSUN. Furthermore, we report prevalence by cancer type, finding significant variation in this need, ranging from 9.4% for individuals ever diagnosed with haematological cancer to 37.8% for individuals ever diagnosed with breast cancer.

Weighted point estimates and how they vary by cancer type provide a basis for stakeholder engagement around priorities for action. For example, prevalence estimates from the SCNS-SF34 showed that other than for prostate and gynaecological cancers, ‘concern for others’, ‘not being able to be the things you used to do’, and ‘energy/tiredness’ are ranked in the top five for all the six other cancer categories. For prostate cancer and gynaecological cancers, ‘changes in sexual relationships’ and ‘fear of spread’ are the top-ranked supportive care need, respectively. Such findings could inform discussions with stakeholders that lead to the prioritisation of defined steps to assess the feasibility, acceptability, and value of general and cancer-specific interventions that address specific supportive care needs.

To further inform and support stakeholder engagement, preliminary literature searches could be undertaken to identify existing evidence on interventions that address the top-ranked supportive care needs. A recently published review of reviews of supportive care needs interventions provides a starting resource for such preliminary reviews [23]. As an example, in the current review, energy and tiredness were listed as a prevalent need across cancer types, though they were included in the physical and daily living need domain in the SCNS-SF34 (‘energy/tiredness’), in the emotional need domain in the SUNS (‘dealing with tiredness’) and in the physical and psychological need domain in the SCNAT-IP (‘feeling tired (e.g. sleeping ok)’). The review of reviews identified 99 reviews of interventions to address fatigue, primarily in patients with any type of cancer. There were also reviews in patients with specific cancers, including 16 reviews in breast cancer, four in prostate cancer, two in lung cancer, and single reviews for several other cancers. Most reviews focused on exercise interventions, whilst others included acupuncture, Chinese medicine, complementary and alternative medicines, diet, psychological therapy, expressive writing, art therapy, music therapy, massage, education, and pharmacological interventions [23].

From this review, stakeholders may identify the supportive care needs with the highest unmet needs and then, using the previously published review of reviews, identify a range of potential interventions to address these needs [23]. Following a similar process to that used by Gray et al. [36], stakeholders can then be supported through a process of selecting interventions that are identified as being feasible and acceptable for their local context, which could range from a specific local health service through to local health networks or national service provision, and for which evidence of effectiveness is expected to be transferable. A local economic evaluation of the selected interventions can then reflect their local context, including population characteristics, prevalence of cancer and unmet needs, cost and effectiveness estimates of proposed interventions, and information on existing services. Again, stakeholders can be engaged to support the conduct of an economic evaluation to deliver health services with the greatest net health benefit for their local context [37].

Comparing our findings with country-specific international literature presents challenges due to limited available data. Apart from the Lisy et al. [14] review discussed earlier in this manuscript, we could only find two systematic reviews that specifically aimed to assess the prevalence of unmet needs within specific countries. The first review investigated the supportive care needs of Chinese patients, differentiating between native and immigrant backgrounds [38]. The study analysed 45 studies and found that the most common unmet need for native and immigrant Chinese individuals fell within the health system and information domain, followed by psychological needs. The second review focused on unmet needs among Japanese patients but included only five studies involving adults [39]. The review found that the psychological domain had the highest unmet needs, followed by information needs. It is worth noting that both these studies reported only the number of studies in which the item or domain was endorsed and the range of reported prevalence. Given the potential impact of different healthcare models on patients’ unmet needs, we recommend conducting similar reviews in other countries to facilitate informed service prioritisation.

### Comparison across tools

Whilst there is a general pattern of high unmet psychological needs, there were variations in the prevalence of these needs between studies using different assessment tools. When considering all the studies collectively, those using the SCNS-SF34 tool tend to report a higher prevalence of unmet psychological needs than those using the CaSUN tool. When considering specific cancer types, we observed variations in the estimated prevalence of the top-ranked unmet supportive care need, depending on whether the CaSUN or the SCNS-SF34 tools were used. For individuals ever diagnosed with breast cancer or those with mixed cancer types, studies using CaSUN estimated a higher prevalence (37.8% and 35.2%) than those using SCNS-SF34 (19.9% and 22.4%). Conversely, for brain, pancreatic, and gynaecological cancers, studies using SCNS-SF34 reported a higher prevalence (47.1%, 40.6%, and 36.3%) in comparison to CaSUN (26.4%, not available, and 13.0%). This suggests that different assessment tools may capture needs more salient to specific cancer types or individuals at various points on the care continuum. Alternatively, these differences could result from methodological distinctions among the primary studies, potentially influenced by response bias. These differences could influence decisions related to service prioritisation. For example, our review identified a similar top unmet need among individuals ever diagnosed with gynaecological cancer, but with varying prevalence estimates: ‘concerns about the cancer coming back’ was reported at 13.0% when using CaSUN, whilst ‘fears about the cancer spreading’ had a prevalence of 36.3% using SCNS-SF34. The choice between using prevalence estimates from CaSUN or SCNS-SF34 may have implications for cost-effectiveness and budget impact analyses of interventions targeting this unmet need. It is important to note that both tools have demonstrated their reliability and validity [40], and the prevalence proportions were estimated from three or more studies with over 800 participants each (SCNS-SF34, *n* = 997; CaSUN, *n* = 840). In this situation, decision-makers have two options: they can review the primary studies to determine which ones best reflect the patient population relevant to the proposed intervention within their local context, or they can conduct sensitivity analyses to assess the cost-effectiveness across varying prevalence proportions.

The SCNAT-IP tool, specifically developed for and by Aboriginal and Torres Strait Islander populations [41], can provide valuable insights into the unique experiences, needs, and outcomes of Aboriginal and Torres Strait Islander cancer patients. The high incidence and mortality rates of cancer among Aboriginal and Torres Strait Islanders, as well as their lower cancer survival rates [42], highlight the importance of tailoring healthcare interventions to meet the specific needs of this population. Therefore, the inclusion of culturally specific patient-reported measures like the SCNAT-IP is crucial for providing equitable and effective cancer care for Aboriginal and Torres Strait Islander populations.

### Limitations

This study has several limitations. First, the conclusions of this review are limited by the range of primary studies available for inclusion. For example, no primary studies assessing the unmet supportive care needs of individuals ever diagnosed with rectal or kidney cancer met our inclusion criteria despite having a higher age-standardised prevalence in Australia than pancreatic or brain cancer [43]. Aggregated results are, therefore, influenced by the needs of individuals ever diagnosed with the types of cancer included in the review. Whilst we present estimates weighted by the sample size of the primary study, we do not consider the proportional prevalence of cancer type in Australia. This additional input should be considered by stakeholders in prioritising services to address needs. Similarly, conclusions of prevalent needs are limited by the items included in the tools. For example, not all tools include all items, such as accessibility of hospital parking, and some more recently identified needs, such as concern with cognitive issues [44], are not included. Second, this review makes assumptions on prevalence where complete data was unavailable for all items from the primary studies. Whilst every effort was made to contact the authors to obtain this additional information (complete data was received from 48% of those contacted), for items where prevalence was not available, we assumed that the prevalence was 50% lower than the lowest reported item in the respective tool. The conventional approach in other systematic reviews has been to report the number of studies in which the item was endorsed and the range of prevalences reported. However, we found this approach had limitations regarding its utility for service prioritisation, as it often resulted in wide prevalence ranges. Additionally, these reviews tended to assume that unreported unmet need items were not endorsed by respondents, introducing a potential bias. Given that our primary focus in this manuscript is to demonstrate how the prevalence of unmet needs can inform service prioritisation, we aimed to develop a method to provide more precise point prevalence estimates. Therefore, we consider our approach more appropriate than assuming these items had zero prevalence. Importantly, sensitivity analyses presenting an upper (assumed prevalence at 0.1% below the lowest reported prevalence) and lower (assumed prevalence at 0) bound on potential prevalence estimates did not change the substantive interpretation of the results. Third, we could not compare the needs of patients currently undergoing treatment with those who had completed treatment as the primary studies reported on the period since diagnosis and treatment received rather than treatment status. Fourth, we could not investigate trends due to the heterogeneity in the primary studies. Lastly, it is important to recognise the impact of our decision to focus the review exclusively on studies employing one of the four generic unmet needs tools. Whilst this choice was essential for enabling point prevalence estimates, it resulted in the exclusion of a significant number of papers. This exclusion may have implications for the generalisability and comprehensiveness of our findings.

## Conclusions

This review examines the prevalence of unmet needs for individuals ever diagnosed with cancer across various cancer types. Our findings confirm the high prevalence of unmet supportive care needs for Australians ever diagnosed with cancer, underscoring the necessity for improved supportive care services. Notably, psychological supportive care needs emerge as the most prevalent across all cancer types, highlighting the importance of prioritising interventions in this domain. In addition, this review provides valuable point prevalence estimates for specific unmet supportive care needs across all cancers and within particular cancer types. These estimates can be combined with cost and effectiveness data to guide the prioritisation of interventions addressing supportive care needs for cancer survivors.

### Supplementary information

ESM 1: (DOCX 59.9 kb)

## Data Availability

The authors confirm that the data supporting the findings of this study are available within the article and its supplementary materials.
