# Supplementary material for: Prevalence of unmet supportive care needs reported by individuals ever diagnosed with cancer in Australia: a systematic review to support service prioritisation
Source: Support Care Cancer. 2023 Nov 7;31(12):676. doi: 10.1007/s00520-023-08146-y (PMC10630245; doi:10.1007/s00520-023-08146-y)
Supplement: Supplementary file 1 — (DOCX 59.9 kb) [file 520_2023_8146_MOESM1_ESM.docx]

# Supplementary Material

## Supplementary Table 1: Medline search strategy

**MEDLINE** 18.11.2020

| **Concept** | **#** | **Search** | **MEDLINE** |
| --- | --- | --- | --- |
| Cancer | 1 | exp Neoplasms/ | 3,384,930 |
|  | 2 | (cancer* or neoplas* or tumor* or tumour* or malignan* or carcinoma* or metasta* or oncolog* or leukemi* or leukaemi* or lymphoma* or myeloma* or sarcoma*).ti,kf. | 2,586,985 |
|  | 3 | or/1-2 | 3,857,985 |
| General needs | 4 | Needs Assessment/ OR exp "Health Services Needs and Demand"/ | 89,082 |
|  | 5 | ((supportive care) OR (need* assess*) OR (needs assess*) OR (patient* need*) OR (support* care need*) OR (support* need*) OR (supportive therap*) OR (unmet need*) OR (healthcare need*) OR (health concern*) OR (need for support*) OR (symptom management) OR (care need*) OR (perceived need*) OR (health service* need*)).tw,kf. | 123,551 |
|  | 6 | or/4-5 | 199,873 |
| Financial | 7 | COST OF ILLNESS/ | 27,806 |
|  | 8 | ((cost* or financ* or unemploy* or employ* or work*) adj2 (toxicity or distress or stress or strain or hardship or advice or need* or assistance or barrier* or impact or burden* or effect or worry or worries or support or poverty or difficult* OR problem* or consequence* or income)).tw,kf | 96,070 |
|  | 9 | ("out-of-pocket cost*" or "out-of-pocket expen*" OR "out-of-pocket payment*" OR "out-of-pocket spend*" OR co-pay*).tw,kf. | 3,402 |
|  | 10 | or/7-9 | 123,853 |
| Intimacy | 11 | ((Reproduct* or sex*) adj (behavio?r or function OR expression OR orientation OR belief* OR intimacy OR intimate OR intercourse OR health)).tw,kf. | 83,149 |
|  | 12 | Sexual Abstinence/ OR Sexuality/ OR Sexual Partners/ | 25,204 |
|  | 13 | (Abortion, induced OR termination of pregnancy).tw,kf. | 11,580 |
|  | 14 | Sexually Transmitted Diseases/ | 24,829 |
|  | 15 | (Sexually transmitted adj (infection* or disease*)).tw,kf. | 30,956 |
|  | 16 | orgasm/ OR libido/ | 6,712 |
|  | 17 | (orgasm* OR libido OR coitus).tw,kf. | 10,762 |
|  | 18 | Reproductive Rights/ | 952 |
|  | 19 | Sexual Dysfunctions, Psychological/ OR Sexual Dysfunction, Physiological/ | 13,854 |
|  | 20 | Dyspareunia/ OR Impotence/ | 20,438 |
|  | 21 | (sexual dysfunction OR impotenc* OR dyspareunia).tw,kf. | 20,290 |
|  | 22 | Or/11-21 | 182,116 |
| Body image | 23 | Body Image/ OR Body Dysmorphic Disorders/ OR ((body dysmorphic disorder*) OR  dysmorphophobia OR (body image)).tw,kf. | 23,537 |
|  | 24 | ((body OR weight OR shape OR physique OR body image) **adj3** (dissatisf* OR satisf* OR disturbance* OR concern* OR preoccupation OR attitude* OR esteem OR appreciation OR awareness OR perception* OR anxiety OR conscious* OR acceptance OR coping OR dysphoria OR avoidance OR (interoceptive awareness) OR self-objectification OR surveillance OR appearance OR delusion OR flexibility OR inflexibility OR functionality OR self-concept OR issue* OR evaluation OR altered OR abnormal)).tw,kf. | 32,406 |
|  | 25 | ((appearance) **adj3** (dissatisf* OR satisf* OR disturbance* OR concern* OR preoccupation OR attitude* OR esteem OR appreciation OR awareness OR perception* OR anxiety OR conscious* OR acceptance OR coping OR dysphoria OR avoidance OR (interoceptive awareness) OR self-objectification OR surveillance OR delusion OR flexibility OR inflexibility OR functionality OR self-concept OR issue* OR evaluation OR altered OR abnormal)).tw,kf. | 5,936 |
|  | 26 | Cicatrix/ OR (disfigure* OR (visible difference) OR scar*).tw,kf. | 179,239 |
|  | 27 | 23 OR 24 OR 25 OR 26 | 229,544 |
|  | 28 | exp Malnutrition/ OR exp Nutritional Status/ | 159,899 |
| Dietary | 29 | (((dietary guideline*) OR diet* OR food OR eat* OR nutri* OR weight OR energy OR (energy intake)) **adj3** (concern* OR need* OR dissatis* OR satisf* OR perception* OR anxiety OR acceptance OR coping OR surveillance OR issue* OR problem* OR regime)).tw,kf. | 48,712 |
|  | 30 | 28 OR 29 | 204,377 |
| Physical and daily living | 31 | "Activities of Daily Living"/ OR *Exercise/ | 141,500 |
|  | 32 | ((physical OR function* OR mobility OR walking OR ambulat*)  **adj2**  (disabilit* OR abilit* OR limit* OR difficult* OR wellbeing OR 'well being' OR well-being)  ).tw,kf.  OR  ((physical)  **Adj2**  (function*)).tw,kf. | 95,586 |
|  | 33 | ((activities of daily life) OR (activities of daily living*) OR (daily living activities)).tw,kf. | 30,141 |
|  | 34 | 31 OR 32 OR 33 | 236,707 |
| Health system information / education | 35 | ((communicat* OR inform* OR educ*) **adj2** (concern* OR need* OR anxiety OR issue* OR problem*)).tw,kf. | 61,733 |
| Religion | 36 | "Religion and Medicine"/ or Religion/ or exp "Religion and Psychology"/ | 38,963 |
|  | 37 | (religio* OR spiritual* OR miracle OR faith OR god OR prayer*)  **adj3**  (care OR healing OR experience* OR need* OR distress OR (well being) OR well-being OR wellbeing OR belief* OR value* OR practice* OR problem* OR coping OR support* OR commitment OR concern*).tw,kf. | 17,641 |
|  | 38 | 36 OR 37 | 48,437 |
| Psychosocial | 39 | "Quality of Life"/ OR (quality of life).tw,kf. | 199,782 |
|  | 40 | ((emotion* OR social OR psychological OR cogniti* OR relational OR famil* OR marital OR psychosocial OR psycho-social OR mental health OR psychiatric* OR relaxation OR psycho-analytic OR psychoanalytic)  **adj3**  (wellbeing OR (well being) OR well-being OR function* OR difficult* OR distress OR dysfunction* OR support OR (quality of life) OR quality-of-life OR QOL or (health related quality of life) OR (health-related quality of life) OR (health related quality-of-life) OR (health-related quality-of-life) OR HQOL OR HRQOL OR need* OR concern* OR issue*)).tw,kf. | 340,765 |
|  | 41 | Depression/ OR Stress, psychological/ OR exp Anxiety/ | 283,080 |
|  | 42 | (depression OR stress* OR distress OR anxiety OR anxious* OR depress* OR fear of recurrence).tw,kf. | 1,459,018 |
|  | 43 | or/39-42 | 1,884,283 |
| FINAL  Generic scn + domains | 44 | 6 or 10 or 22 or 27 or 30 or 34 or 35 or 38 or 43 | 2,991,104 |
| FINAL  Cancer + generic scn + domains | 45 | 3 AND 44 | 246,979 |
| Australia* | 46 | exp Australia/ OR (Australia* OR (New South Wales) OR (Northern Territor*) OR Queensland OR (South Australia) OR Tasmania OR Victoria OR (Western Australia) OR Canberra OR Sydney OR Darwin OR Cairns OR Brisbane OR Adelaide OR Hobart OR Melbourne OR Perth).tw,kf,ia. | 220,834 |
| FINAL  Cancer + generic scn + domains + Australia | 47 | 45 AND 46 | 2,865 |
| Time limit – 2010 to current | 48 | limit 47 to (english language and humans and yr="2010 -Current") | 1,789 |

## Supplementary Table 2. Quality Assessment Criteria^^^

| Methodology and procedure | 1. Inclusion/ exclusion criteria are formulated 2. A standardised or valid unmet needs measure is used 3. Participation and response rates for patient groups must be described and must be more than 75% 4. Patient signed an informed consent form before study participation |
| --- | --- |
| Information about cancer survivors | 1. Socio-economic data are described (e.g., age, race, employment status, educational status 2. Mean or median and range or standard deviation of time since diagnosis or treatment is given 3. Information is presented about cancer stage at diagnosis 4. The type of cancer treatment is described |
| Results and interpretation | 1. Results are presented for the total unmet needs 2. Results are presented for each domain separately 3. An attempt is made to find factors associated with higher unmet needs* 4. Authors were aware of their limitations |

* Excluded as not relevant to our study

^^^Tool from Miroševič et al. (1)

## Supplementary Table 3: Quality Assessment

|  | **Methodology and procedure** | | | | **Information about cancer survivors** | | | | | **Results and interpretation** | | | | **Score** |
| --- | --- | --- | --- | --- | --- | --- | --- | --- | --- | --- | --- | --- | --- | --- |
|  | **Q1** | **Q2** | **Q3** | **Q4** | | **Q5** | **Q6** | **Q7** | **Q8** | | **Q9** | **Q10** | **Q12** |  |
| Ahern (2016) | Y | Y | N | Y | | Y | N | N | Y | | N | N | Y | 6 |
| Alananzeh (2019) | Y | Y | N | N | | Y | Y | Y | Y | | N | Y | Y | 8 |
| Amatya (2014) | Y | Y | N | Y | | Y | Y | Y | Y | | Y | Y | Y | 10 |
| Beesley (2013) | Y | Y | N | Y | | Y | N | Y | Y | | Y | N | N | 7 |
| Beesley (2016) | Y | Y | N | Y | | Y | Y | Y | Y | | Y | Y | Y | 10 |
| Beesley (2018) | Y | Y | N | Y | | Y | Y | Y | Y | | Y | Y | Y | 10 |
| Bernardes (2019) | Y | Y | Y | Y | | Y | N | Y | Y | | Y | Y | Y | 10 |
| Blaschke (2019) | Y | Y | N | Y | | Y | Y | Y | Y | | N | N | Y | 8 |
| Boyes (2012) | Y | Y | N | Y | | Y | Y | Y | Y | | Y | Y | Y | 10 |
| Boyes (2015) | Y | Y | N | Y | | Y | Y | N | Y | | Y | Y | Y | 9 |
| Brennan (2016) | Y | Y | Y | Y | | N | N | Y | Y | | Y | N | Y | 8 |
| Chambers (2012) | Y | Y | N | Y | | Y | Y | N | N | | N | N | Y | 6 |
| Dunn (2022) | Y | Y | N | Y | | Y | Y | N | N | | N | N | Y | 6 |
| Eggins (2022) | Y | Y | N | Y | | Y | N | N | N | | N | Y | Y | 6 |
| Garvey (2015) | Y | Y | Y | Y | | Y | Y | Y | Y | | Y | N | Y | 10 |
| Gough (2022) | Y | Y | N | Y | | Y | N | Y | Y | | N | N | Y | 7 |
| Hall (2012) | Y | Y | Y | Y | | Y | Y | Y | Y | | N | Y | Y | 10 |
| Hall (2015) | Y | Y | N | Y | | Y | Y | N | N | | N | N | Y | 6 |
| Halkett (2015) | Y | Y | Y | Y | | Y | N | Y | Y | | Y | N | N | 8 |
| Hyde (2017) | Y | Y | N | Y | | Y | Y | N | Y | | Y | Y | Y | 9 |
| Khan (2013) | Y | Y | N | Y | | Y | Y | Y | Y | | Y | N | Y | 9 |
| Kusters (2015) | Y | Y | N | Y | | Y | Y | Y | Y | | N | N | Y | 8 |
| Langbecker (2016) | Y | Y | N | Y | | Y | N | Y | Y | | N | Y | Y | 8 |
| Mazariego (2020) | Y | Y | N | Y | | Y | N | Y | Y | | Y | Y | Y | 9 |
| McDowell (2010) | Y | Y | N | Y | | Y | Y | N | Y | | Y | Y | N | 8 |
| Molassiotis (2017) | Y | Y | N | Y | | Y | Y | N | Y | | N | Y | Y | 8 |
| Oberoi (2016) | Y | Y | N | Y | | Y | Y | N | Y | | N | Y | Y | 8 |
| Rowlands (2015) | Y | Y | N | Y | | Y | N | Y | Y | | Y | N | Y | 8 |
| Smith (2013) | Y | Y | N | Y | | Y | Y | Y | Y | | Y | Y | Y | 10 |
| Stafford (2011) | Y | Y | N | Y | | Y | Y | Y | Y | | Y | N | Y | 9 |
| Tzelepis (2018) | Y | Y | N | Y | | Y | Y | N | Y | | N | Y | Y | 8 |
| Urbaniec (2011) | Y | Y | N | Y | | Y | Y | Y | Y | | Y | N | Y | 9 |
| Vuksanovic (2021) | Y | Y | N | N | | Y | Y | N | Y | | Y | N | Y | 7 |
| Williams (2018) | Y | Y | N | Y | | Y | Y | N | Y | | Y | N | Y | 8 |
| Yates (2021) | Y | Y | N | Y | | Y | N | N | Y | | N | Y | Y | 7 |

## Supplementary Table 4: Prevalence of all 34 unmet supportive care needs as identified by the Supportive Care Needs Survey Short-Form 34 (SCNS-SF34) by cancer type, studies including all cancer types (mixed) and aggregated across all studies

| **Rank** | **Gynaecological  (n=4 studies)** | **Haematological*  (n=2 studies)** | **Brain*  (n=2 studies)** | **Breast*  (n=3 studies)** | **Pancreatic*  (n=1 studies)** | **Neuroendocrine*  (n=1 studies)** | **Prostate  (n=2 studies)** | **Mixed  (n=6 studies)** | **All  (n=21 studies)** |
| --- | --- | --- | --- | --- | --- | --- | --- | --- | --- |
| 1 | Fear of spread (36.3%) | Not being able to do the things you used to do (21.5%) | Concern for others (47.1%) | Energy/tiredness (19.9%) | Not being able to do the things you used to do (40.6%) | Energy/tiredness (32.4%) | Changes in sexual relationships (27.0%) | Concern for others (22.4%) | Fear of spread (20.8%) |
| 2 | Informed about test results (34.8%) | Energy/tiredness (19.9%) | Not being able to do the things you used to do (42.8%) | Fear of spread (19.6%) | Concern for others (37.3%) | Concern for others (26.1%) | Changes in sexual feelings (26.7%) | Fear of spread (22.3%) | Energy/tiredness (20.6%) |
| 3 | Info on self-management (33.1%) | Concern for others (18.5%) | Future uncertainty (36.1%) | Future uncertainty (17.6%) | Future uncertainty (30.4%) | Fear of spread (26.1%) | Concern for others (14.9%) | Future uncertainty (22.3%) | Concern for others (20.4%) |
| 4 | Hospital staff - one contact point (32.5%) | Work around the home (16.3%) | Energy/tiredness (34.6%) | Not being able to do the things you used to do (17.1%) | Work around the home (28.4%) | Future uncertainty (25.2%) | Not being able to do the things you used to do (13.3%) | Not being able to do the things you used to do (21.7%) | Not being able to do the things you used to do (20.1%) |
| 5 | Info on remission (32.3%) | Future uncertainty (14.6%) | Depressed/down (27.9%) | Concern for others (16.1%) | Energy/tiredness (28.2%) | Not being able to do the things you used to do (23.6%) | Fear of spread (12.1%) | Energy/tiredness (20.8%) | Future uncertainty (19.9%) |
| 6 | Treated like a person (32.2%) | Learning to feel in control (11.2%) | Anxiety (27.8%) | Anxiety (15.1%) | Fear of spread (25.7%) | Depressed/down (18.9%) | Info on sexual relationships (11.8%) | Worry about results (19.4%) | Worry about results (16.4%) |
| 7 | Concern for others (32.0%) | Positive outlook (10.8%) | Informed about test results (27.1%) | Depressed/down (14.7%) | Pain (25.7%) | Work around the home (17.4%) | Future uncertainty (11.7%) | Work around the home (16.0%) | Sadness (15.5%) |
| 8 | Info on treatment benefits and side effects (31.5%) | Feeling unwell (10.7%) | Info on self-management (24.8%) | Sadness (13.9%) | Worry about results (23.1%) | Anxiety (17.3%) | Info on self-management (11.2%) | Anxiety (15.9%) | Depressed/down (15.4%) |
| 9 | Future uncertainty (29.4%) | Sadness (10.4%) | Sadness (24.5%) | Worry about results (13.9%) | Learning to feel in control (21.5%) | Sadness (17.1%) | Depressed/down (10.7%) | Depressed/down (15.3%) | Anxiety (15.1%) |
| 10 | Energy/tiredness (28.6%) | Fear of spread (9.4%) | Info on remission (23.8%) | Changes in sexual feelings (13.4%) | Positive outlook (20.9%) | Worry about results (17.1%) | Hospital staff - one contact point (10.5%) | Sadness (14.5%) | Learning to feel in control (14.5%) |
| 11 | Explanations for tests (28.5%) | Depressed/down (9.4%) | Positive outlook (23.5%) | Changes in sexual relationships (12.6%) | Feeling unwell (20.0%) | Learning to feel in control (16.2%) | Energy/tiredness (9.5%) | Learning to feel in control (14.4%) | Work around the home (14.2%) |
| 12 | Pleasant hospital/clinic (28.3%) | Worry about results (8.6%) | Learning to feel in control (23.5%) | Learning to feel in control (12.0%) | Info on self-management (18.9%) | Pain (15.7%) | Anxiety (9.4%) | Pain (13.2%) | Changes in sexual feelings (14.0%) |
| 13 | Access to counselling (26.9%) | Pain (7.7%) | Fear of spread (22.9%) | Work around the home (11.8%) | Hospital staff - one contact point (17.4%) | Info on self-management (15.6%) | Info on treatment benefits and side effects (9.2%) | Changes in sexual feelings (11.5%) | Pain (13.2%) |
| 14 | Worry about results (26.6%) | Feelings about death and dying (6.9%) | Work around the home (22.8%) | Pain (11.6%) | Anxiety (16.9%) | Changes in sexual feelings (13.6%) | Worry about results (8.9%) | Info on self-management (11.3%) | Changes in sexual relationships (12.8%) |
| 15 | Anxiety (25.9%) | Access to counselling (6.4%) | Worry about results (22.4%) | Access to counselling (10.5%) | Access to counselling (16.0%) | Feeling unwell (13.1%) | Access to counselling (8.2%) | Changes in sexual relationships (11.3%) | Info on self-management (12.6%) |
| 16 | Info on care (25.1%) | Changes in sexual feelings (6.0%) | Hospital staff - one contact point (21.1%) | Hospital staff - one contact point (10.3%) | Explanations for tests (15.9%) | Positive outlook (12.7%) | Sadness (7.6%) | Feeling unwell (10.9%) | Hospital staff - one contact point (12.6%) |
| 17 | Not being able to do the things you used to do (24.8%) | Changes in sexual relationships (6.0%) | Info on treatment benefits and side effects (19.2%) | Positive outlook (9.7%) | Info on managing side effects (15.9%) | Informed about test results (12.7%) | Learning to feel in control (6.9%) | Informed about test results (10.2%) | Access to counselling (12.1%) |
| 18 | Info on managing side effects (24.4%) | Anxiety (6.0%) | Feelings about death and dying (19.1%) | Feelings about death and dying (9.0%) | Depressed/down (15.4%) | Changes in sexual relationships (11.7%) | Informed about test results (6.6%) | Hospital staff - one contact point (10.1%) | Positive outlook (11.7%) |
| 19 | Hospital staff - emotional needs (23.5%) | Informed about test results (6.0%) | Info on care (19.1%) | Info on self-management (9.0%) | Info on remission (15.0%) | Feelings about death and dying (10.9%) | Info on remission (6.6%) | Positive outlook (10.0%) | Info on remission (11.1%) |
| 20 | Learning to feel in control (23.4%) | Hospital staff - one contact point (6.0%) | Access to counselling (19.0%) | Feeling unwell (7.8%) | Sadness (14.7%) | Info on remission (10.2%) | Explanations for tests (6.3%) | Info on remission (9.8%) | Informed about test results (10.7%) |
| 21 | Positive outlook (23.0%) | Info on managing side effects (5.6%) | Changes in sexual relationships (18.6%) | Info on remission (7.6%) | Informed about test results (14.3%) | Hospital staff - one contact point (10.0%) | Info on managing side effects (5.8%) | Access to counselling (9.7%) | Info on treatment benefits and side effects (10.3%) |
| 22 | Depressed/down (22.5%) | Info on remission (5.6%) | Info on managing side effects (17.9%) | Info on treatment benefits and side effects (7.1%) | Feelings about death and dying (14.1%) | Treated like a person (9.1%) | Choice - specialists (5.6%) | Info on treatment benefits and side effects (9.7%) | Feelings about death and dying (10.3%) |
| 23 | Sadness (21.1%) | Info on self-management (5.6%) | Pleasant hospital/clinic (17.1%) | Informed about test results (6.8%) | Info on treatment benefits and side effects (13.5%) | Info on sexual relationships (8.3%) | Feeling unwell (5.5%) | Feelings about death and dying (9.4%) | Feeling unwell (10.0%) |
| 24 | Medical staff - reassurance (20.8%) | Explanations for tests (5.1%) | Changes in sexual feelings (16.9%) | Treated like a person (6.6%) | Treated like a person (12.8%) | Info on managing side effects (8.2%) | Treated like a person (5.2%) | Explanations for tests (9.0%) | Treated like a person (10.0%) |
| 25 | Work around the home (20.4%) | Pleasant hospital/clinic (5.1%) | Treated like a person (16.5%) | Info on sexual relationships (6.1%) | Pleasant hospital/clinic (12.0%) | Info on treatment benefits and side effects (7.3%) | Info on care (4.9%) | Info on managing side effects (8.9%) | Info on managing side effects (9.4%) |
| 26 | Changes in sexual feelings (19.2%) | Hospital staff - emotional needs (4.7%) | Pain (16.4%) | Medical staff - reassurance (6.0%) | Changes in sexual relationships (10.6%) | Info on care (7.3%) | Choice - hospital (4.8%) | Treated like a person (8.5%) | Explanations for tests (9.3%) |
| 27 | Pain (18.9%) | Hospital staff - physical needs (4.7%) | Explanations for tests (15.4%) | Explanations for tests (5.5%) | Choice - hospital (10.5%) | Access to counselling (7.3%) | Positive outlook (4.2%) | Info on sexual relationships (8.4%) | Info on care (8.9%) |
| 28 | Feelings about death and dying (18.8%) | Medical staff - reassurance (4.7%) | Choice - specialists (14.6%) | Info on managing side effects (5.5%) | Hospital staff - emotional needs (9.9%) | Pleasant hospital/clinic (6.4%) | Feelings about death and dying (3.9%) | Info on care (8.3%) | Medical staff - reassurance (8.4%) |
| 29 | Hospital staff - physical needs (17.8%) | Info on treatment benefits and side effects (4.7%) | Feeling unwell (13.8%) | Pleasant hospital/clinic (5.3%) | Info on care (9.9%) | Medical staff - reassurance (6.3%) | Medical staff - reassurance (3.9%) | Pleasant hospital/clinic (7.9%) | Info on sexual relationships (8.2%) |
| 30 | Changes in sexual relationships (17.5%) | Info on care (4.7%) | Choice - hospital (13.2%) | Info on care (4.9%) | Hospital staff - physical needs (9.7%) | Choice - specialists (5.5%) | Pain (3.6%) | Medical staff - reassurance (7.8%) | Pleasant hospital/clinic (7.9%) |
| 31 | Info on sexual relationships (15.7%) | Treated like a person (4.7%) | Hospital staff - physical needs (12.6%) | Choice - specialists (4.7%) | Changes in sexual feelings (9.0%) | Choice - hospital (3.6%) | Work around the home (3.1%) | Choice - specialists (7.2%) | Choice - specialists (7.0%) |
| 32 | Feeling unwell (14.9%) | Info on sexual relationships (4.3%) | Info on sexual relationships (12.2%) | Hospital staff - emotional needs (4.5%) | Choice - specialists (9.0%) | Explanations for tests (3.6%) | Hospital staff - physical needs (2.6%) | Hospital staff - emotional needs (7.1%) | Hospital staff - physical needs (6.7%) |
| 33 | Choice - specialists (12.8%) | Choice - hospital (4.3%) | Medical staff - reassurance (12.0%) | Hospital staff - physical needs (4.4%) | Medical staff - reassurance (8.3%) | Hospital staff - emotional needs (3.6%) | Pleasant hospital/clinic (2.3%) | Hospital staff - physical needs (6.8%) | Choice - hospital (6.6%) |
| 34 | Choice - hospital (11.3%) | Choice - specialists (3.9%) | Hospital staff - emotional needs (11.9%) | Choice - hospital (3.5%) | Info on sexual relationships (6.8%) | Hospital staff - physical needs (2.7%) | Hospital staff - emotional needs (2.1%) | Choice - hospital (6.6%) | Hospital staff - emotional needs (6.5%) |

* Note the jointly ranked items by prevalence.

Items are colour-coded by domains. SCNS-SF has five domains

|  | Physical and daily living needs |  | Health system and information needs |  | Sexual needs |  | Psychological needs |  | Patient care & support |
| --- | --- | --- | --- | --- | --- | --- | --- | --- | --- |

## Supplementary Table 5: Prevalence of all 35 unmet supportive care needs as identified by the Cancer Survivors’ Unmet Needs (CaSUN) tool by cancer type, studies including all cancer types (mixed) and aggregated across all studies

| **Rank** | **Brain*  (n=2 studies)** | **Breast  (n=3 studies)** | **Prostate*  (n=1 studies)** | **Testicular*  (n=1 studies)** | **Gynaecological*  (n=3 studies)** | **Mixed  (n=1 studies)** | **All  (n=11 studies)** |
| --- | --- | --- | --- | --- | --- | --- | --- |
| 1 | Decisions about my life (26.4%) | Concerns about the cancer coming back (37.8%) | Accessible hospital parking (15.1%) | Reduce stress in my life (30.1%) | Concerns about the cancer coming back (13.0%) | Accessible hospital parking (35.2%) | Concerns about the cancer coming back (17.9%) |
| 2 | Concerns about the cancer coming back (24.5%) | Reduce stress in my life (29.5%) | Doctors talk to each other (14.0%) | Problems with sex life (23.3%) | Reduce stress in my life (12.4%) | Doctors talk to each other (15.9%) | Reduce stress in my life (17.2%) |
| 3 | Reduce stress in my life (23.6%) | Changes to my body (24.1%) | Problems with sex life (13.4%) | Financial support (21.8%) | Doctors talk to each other (11.2%) | Complimentary therapy (11.2%) | Accessible hospital parking (15.0%) |
| 4 | Talk to others (23.6%) | Complimentary therapy (23.9%) | Complaints addressed (11.7%) | Concerns about the cancer coming back (21.7%) | Acknowledging the impact (11.0%) | Life/travel insurance (10.9%) | Doctors talk to each other (12.9%) |
| 5 | Accessible hospital parking (21.7%) | Doctors talk to each other (23.7%) | Manage health with team (10.0%) | Life/travel insurance (20.4%) | Decisions about my life (9.8%) | Concerns about the cancer coming back (10.1%) | Problems with sex life (11.8%) |
| 6 | Support partner/family (17.0%) | Manage side effects (23.3%) | Understandable information (9.7%) | Acknowledging the impact (17.5%) | Emotional support for me (9.3%) | Financial support (10.0%) | Decisions about my life (11.0%) |
| 7 | Complimentary therapy (17.0%) | Accessible hospital parking (22.6%) | Local health care services (9.7%) | Accessible hospital parking (17.2%) | Complaints addressed (8.7%) | Reduce stress in my life (9.7%) | Acknowledging the impact (10.6%) |
| 8 | Financial support (16.0%) | Manage health with team (19.9%) | Manage side effects (9.7%) | Talk to others (17.1%) | Complimentary therapy (8.6%) | Up to date information (8.6%) | Complimentary therapy (10.3%) |
| 9 | Changes to quality of life (15.1%) | Ongoing case manager (19.5%) | Concerns about the cancer coming back (9.7%) | Survivor expectations (17.1%) | Problems with sex life (8.3%) | Ongoing case manager (8.6%) | Complaints addressed (10.3%) |
| 10 | Changes to my body (14.2%) | Changes to quality of life (17.9%) | Up to date information (9.1%) | Emotional support for me (16.7%) | Accessible hospital parking (7.6%) | Complaints addressed (8.0%) | Emotional support for me (10.0%) |
| 11 | Ongoing case manager (14.2%) | Complaints addressed (17.7%) | Best medical care (9.1%) | Up to date information (8.4%) | Ongoing case manager (7.4%) | Local health care services (7.8%) | Changes to my body (9.9%) |
| 12 | Up to date information (7.5%) | Up to date information (17.3%) | Changes to quality of life (8.9%) | Information for others (8.4%) | Survivor expectations (7.1%) | Information for others (7.7%) | Manage side effects (9.7%) |
| 13 | Information for others (7.5%) | Understandable information (16.7%) | Impact on my relationship (8.9%) | Understandable information (8.4%) | Manage health with team (6.8%) | Decisions about my life (7.4%) | Manage health with team (9.4%) |
| 14 | Understandable information (7.5%) | Problems with sex life (16.0%) | Reduce stress in my life (8.6%) | Best medical care (8.4%) | Changes to my body (6.5%) | Make my life count (7.4%) | Talk to others (9.2%) |
| 15 | Best medical care (7.5%) | Survivor expectations (15.8%) | Information for others (8.0%) | Local health care services (8.4%) | Local health care services (6.4%) | Understandable information (6.7%) | Survivor expectations (8.9%) |
| 16 | Local health care services (7.5%) | Decisions about my life (15.1%) | Changes to my body (6.6%) | Manage health with team (8.4%) | Manage side effects (6.4%) | Best medical care (6.7%) | Ongoing case manager (8.8%) |
| 17 | Manage health with team (7.5%) | Emotional support for me (15.0%) | Emotional support for me (5.7%) | Doctors talk to each other (8.4%) | Best medical care (5.8%) | Manage health with team (6.7%) | Changes to quality of life (8.7%) |
| 18 | Doctors talk to each other (7.5%) | Acknowledging the impact (15.0%) | Acknowledging the impact (5.1%) | Complaints addressed (8.4%) | Changes to beliefs (5.0%) | Manage side effects (6.6%) | Financial support (8.6%) |
| 19 | Complaints addressed (7.5%) | Financial support (14.2%) | Talk to others (4.6%) | Manage side effects (8.4%) | Talk to others (4.9%) | Changes to quality of life (6.5%) | Local health care services (8.5%) |
| 20 | Manage side effects (7.5%) | Local health care services (14.1%) | Move on with my life (4.6%) | Changes to quality of life (8.4%) | Up to date information (4.3%) | Emotional support for me (6.5%) | Up to date information (7.9%) |
| 21 | Emotional support for me (7.5%) | Information for others (13.5%) | Support partner/family (4.6%) | New relationships (8.4%) | Information for others (4.3%) | Talk to others (6.5%) | Understandable information (7.9%) |
| 22 | New relationships (7.5%) | Best medical care (12.6%) | New relationships (4.3%) | Handle social/work situations (8.4%) | Understandable information (4.3%) | Survivor expectations (6.5%) | Best medical care (7.9%) |
| 23 | Handle social/work situations (7.5%) | Changes to beliefs (12.5%) | Changes to beliefs (4.3%) | Changes to my body (8.4%) | Changes to quality of life (4.3%) | Impact on my relationship (5.4%) | Life/travel insurance (7.2%) |
| 24 | Move on with my life (7.5%) | Impact on my relationship (11.5%) | Decisions about my life (4.0%) | Move on with my life (8.4%) | New relationships (4.3%) | Problems with sex life (5.4%) | Information for others (7.2%) |
| 25 | Changes to beliefs (7.5%) | Life/travel insurance (11.3%) | Survivor expectations (3.4%) | Changes to beliefs (8.4%) | Handle social/work situations (4.3%) | Changes to my body (5.3%) | Impact on my relationship (7.0%) |
| 26 | Acknowledging the impact (7.5%) | Move on with my life (11.2%) | Handle social/work situations (2.3%) | Decisions about my life (8.4%) | Move on with my life (4.3%) | Move on with my life (4.3%) | Support partner/family (6.8%) |
| 27 | Survivor expectations (7.5%) | Talk to others (11.2%) | Make my life count (1.7%) | Spiritual beliefs (8.4%) | Spiritual beliefs (4.3%) | Acknowledging the impact (4.3%) | Changes to beliefs (6.5%) |
| 28 | Spiritual beliefs (7.5%) | Support partner/family (9.1%) | Spiritual beliefs (1.1%) | Make my life count (8.4%) | Make my life count (4.3%) | Support partner/family (4.3%) | Move on with my life (6.1%) |
| 29 | Make my life count (7.5%) | Employment (8.6%) | Complimentary therapy (0.6%) | Support partner/family (8.4%) | Support partner/family (4.3%) | New relationships (3.2%) | New relationships (5.4%) |
| 30 | Impact on my relationship (7.5%) | Legal services (8.3%) | Employment (0.6%) | Impact on my relationship (8.4%) | Impact on my relationship (4.3%) | Handle social/work situations (3.2%) | Make my life count (5.4%) |
| 31 | Problems with sex life (7.5%) | Make my life count (8.2%) | Financial support (0.6%) | Complimentary therapy (8.4%) | Employment (4.3%) | Changes to beliefs (3.2%) | Handle social/work situations (5.1%) |
| 32 | Employment (7.5%) | Handle social/work situations (7.4%) | Legal services (0.6%) | Employment (8.4%) | Financial support (4.3%) | Employment (3.2%) | Employment (5.0%) |
| 33 | Legal services (7.5%) | New relationships (7.1%) | Life/travel insurance (0.6%) | Legal services (8.4%) | Legal services (4.3%) | Legal services (3.2%) | Legal services (5.0%) |
| 34 | Life/travel insurance (7.5%) | Spiritual beliefs (6.7%) | Fertility (0.6%) | Fertility (8.4%) | Life/travel insurance (4.3%) | Spiritual beliefs (0.0%) | Spiritual beliefs (4.9%) |
| 35 | Fertility (7.5%) | Fertility (4.0%) | Ongoing case manager (0.6%) | Ongoing case manager (8.4%) | Fertility (4.3%) | Fertility (0.0%) | Fertility (4.6%) |

* Note the jointly ranked items by prevalence.

Items are colour-coded by domains. CaSUN has six domains:

|  | Existential survivorship |  | Quality of Life |  | Relationships |  | Comprehensive cancer care |  | Information |  | Other |
| --- | --- | --- | --- | --- | --- | --- | --- | --- | --- | --- | --- |

## Supplementary Table 6: Prevalence of all 26 unmet supportive care needs as identified by the Supportive Care Needs Assessment Tool for Indigenous People (SCNAT-IP)

| **Rank** | **SCNAT-IP  (n=2 studies)** |
| --- | --- |
| 1 | Financial worry (21.1%) |
| 2 | Illness worry (15.2%) |
| 3 | Family concerns (14.9%) |
| 4 | Anxiety (13.7%) |
| 5 | Usual activities: home (13.4%) |
| 6 | Results worry (13.3%) |
| 7 | Feeling down (12.7%) |
| 8 | Physical pain (11.3%) |
| 9 | Unwell (10.3%) |
| 10 | Cultural support (10.0%) |
| 11 | Usual activities: social (9.8%) |
| 12 | Tired (9.6%) |
| 13 | Accommodation (9.3%) |
| 14 | Information, side effects (9.0%) |
| 15 | Treatment information, home (8.2%) |
| 16 | Treatment information, hospital (7.0%) |
| 17 | Staying positive (6.7%) |
| 18 | Family support (6.2%) |
| 19 | Test explanation (6.2%) |
| 20 | Hospital emotional needs (5.7%) |
| 21 | Self-care (5.5%) |
| 22 | Hospital physical needs (5.2%) |
| 23 | Staff support (4.7%) |
| 24 | Cultural support, communication (4.7%) |
| 25 | Individual care (4.0%) |
| 26 | Hospital navigation (3.6%) |

Items are colour-coded by domains. SCNAT-IP has four domains (no items from: Information and communication; hospital care):

|  | Practical and cultural need |  | Physical and psychological need |  | Information and communication |  | Hospital care |
| --- | --- | --- | --- | --- | --- | --- | --- |

References

1. Miroševič Š, Prins JB, Selič P, Zaletel Kragelj L, Klemenc Ketiš Z. Prevalence and factors associated with unmet needs in post‐treatment cancer survivors: A systematic review. European Journal of Cancer Care. 2019;28(3):e13060.
